# Regulatory risk loci link disrupted androgen response to pathophysiology of Polycystic Ovary Syndrome

**DOI:** 10.1101/2025.03.26.25324630

**Published:** 2025-03-27

**Authors:** Jaya Srivastava, Ivan Ovcharenko

**Affiliations:** Division of Intramural Research, National Library of Medicine, National Institutes of Health, Bethesda, MD, 20892, USA.

**Keywords:** Polycystic Ovary Syndrome (PCOS), regulatory genomics, enhancer variants, deep learning, artificial intelligence, disease-causal noncoding variants

## Abstract

A major challenge in deciphering the complex genetic landscape of Polycystic Ovary Syndrome (PCOS) is the limited understanding of the molecular mechanisms driven by susceptibility loci, necessitating investigation into the regulatory pathways that contribute to the diverse phenotypic manifestations of PCOS. In this study, we integrated molecular and epigenomic annotations across proposed pathogenic cell types and employed a deep learning (DL) model to infer the cell-type-specific effects of risk variants. Our analysis revealed the role of these variants in brain and endocrine cell types affecting the binding sites of key transcription factors (TFs)—*FOXA1, FOXL1, WT1, SALL4*, and *CPEB1*—which regulate ovarian development, folliculogenesis, and steroid hormone signaling, contributing to disease-associated transcriptomic profiles. Our DL model, which is strongly correlated with MPRA data, identified enhancer-disrupting activity in 20% of the risk variants, particularly affecting TFs involved in androgen-mediated signaling, shedding light on the molecular consequences of hyperandrogenemia. Using the *IRX3-FTO* locus as a case study, we explored the potential cell-type-specific regulatory effects of risk variants in the fetal brain, pancreas, adipocytes, and an endothelial cell-line, which suggest that disruptions in IRX3 regulation (previously linked to obesity) may contribute to PCOS pathogenesis through diverse mechanisms, including neuronal development, metabolic regulation, and folliculogenesis. Our findings underscore the value of integrating DL models with epigenomic annotations to identify disease-relevant variants, explore the pleiotropic impact of disease risk loci, and gain novel insights into cross-cell-type regulatory interactions.

## Introduction

Polycystic Ovary Syndrome (PCOS) is a multifactorial endocrine disorder characterized by abnormal LH:FSH (Luteinizing hormone: Follicle Stimulating hormone) ratios and elevated androgen levels, leading to anovulation, polycystic ovaries, and various hyperandrogenism-related comorbidities^1–3^. The reproductive abnormalities in PCOS stem from disruptions in the hypothalamic-pituitary-gonadal (HPG) axis, which also contributes to other conditions like oligomenorrhoea, ovarian insufficiency, infertility, hyper- and hypogonadism, and endometriosis^4^. This overlap in clinical features complicates PCOS diagnosis, prompting the establishment of multiple diagnostic criteria by the NIH, Rotterdam, and the Androgen Excess and PCOS Society. Based on a consensus, diagnosis relies on clear indications of hyperandrogenism and ovulatory dysfunction^3,5^.

Decades of research on molecular mechanisms underlying the disease have identified impaired steroidogenesis and folliculogenesis in theca and granulosa cells (GCs) as key contributors to PCOS development^6^. These pathways are spatiotemporally regulated by LH and FSH, secreted by the pituitary gland in response to hypothalamic Gonadotropin Release Hormone (GnRH) based stimulation^7^. Moreover, PCOS often coincides with hyperinsulinemia, though the molecular origins of the association between the two is still being investigated. Hyperinsulinemia worsens hyperandrogenism by affecting adrenal androgen production and reducing sex hormone-binding globulin (SHBG) levels in the liver^8^ and may also contribute to the metabolic co-morbidities such as obesity, type-II diabetes and liver dysfunction^3,9^.

The genetic basis of PCOS is thought to involve impaired regulation of the HPG axis^10^. Polymorphisms in genes coding for kisspeptin (Kiss1, an upstream regulator of GnRH), GnRH receptor, Anti-Mullerian Hormone (AMH), LH, FSH, and their receptors have been linked to impaired signaling in PCOS patients^11,12^. However, except for *AMHR*, no functional studies directly connect these polymorphisms to the PCOS phenotype^13^. Twin studies suggest an estimated 80% heritability [13], highlighting the need for detailed investigations into the molecular mechanisms underlying PCOS etiology^14^.

PCOS genome-wide association studies (GWAS) across diverse populations have identified novel disease loci, including plausible candidates such as *FSHR, FSHB*, and *LHCGR*, in addition to several others with no direct association with disease phenotypes^10^. Variants in the loci *of THADA, DENND1A, IRF1, FTO* etc., have significant correlations with the disease manifestation but have not been contextually studied. On the other hand, polymorphisms in reproductive hormone receptors, including androgen (AR) and estrogen receptors (*ESR1/2*), have been implicated in PCOS phenotypes^15,16^; however, these associations have not been consistently replicated in GWAS studies. These observations align with the omnigenic model of complex trait regulation, where core genes directly influence the phenotype, while peripheral genes contribute through cell-type-specific regulatory networks^17^. Variants exert their net impact through complex genomic and epigenomic interactions, manifesting as GWAS association signals. This highlights the need for further investigation into the regulatory networks governing phenotypic complexity of PCOS. In this context, several isolated studies offer insights into the involvement of GWAS-identified genes in PCOS pathophysiology^10^. For instance, studies show that *ERBB4* and *GATA4* regulate folliculogenesis^18,19^, *ZNF217* regulates androgen production in theca cells^20^, and *HMGA2* promotes granulosa cell proliferation^21^. Interestingly, some genes exhibit pleiotropy depending on cell-type context; *HMGA2* also regulates adipogenesis^22^, and FSH influences bone density and adipose mass^4^. These findings suggest that PCOS-associated variants across multiple loci impact different cell-types through hitherto unexplored molecular mechanisms, contributing to phenotypic comorbidities.

In this study, we performed a functional assessment of PCOS susceptibility loci by integrating epigenomic data, functional assays, and a deep learning (DL)-based approach to identify causal single nucleotide variants (SNVs) across eleven disease-associated cell types. We further investigated their potential influence on the molecular mechanisms underlying PCOS etiology. This approach facilitated the identification of key transcription factors (TFs) involved in folliculogenesis, androgen-mediated signaling, and ovarian development, whose binding sites are predicted to be disrupted by causal variants. Using the well-characterized regulatory locus of *IRX3*, we demonstrate how DL models combined with prior knowledge of key PCOS TFs can effectively prioritize causal variants.

## Results

### PCOS risk SNVs are enriched in neuroendocrine cell-types and influence gene expression across multiple cell-types

**Figure 1:**
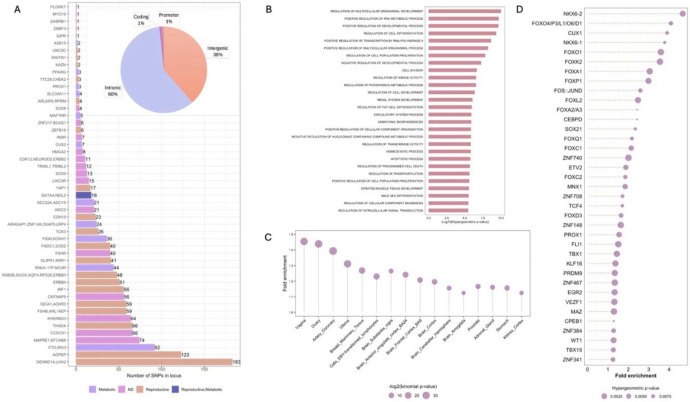
(A) PCOS susceptibility loci and their distribution in non-coding regions, (B) Gene Ontology annotations of target genes, (C) Fold enrichment of PCOS eVariants in GTEx cell-types (p-value < 0.01), (D) Fold enrichment of TFBSs in pcosSNVs compared with other variants in the susceptibility loci.

To conduct a comprehensive analysis of the regulatory features of PCOS risk loci, we identified 91 single nucleotide variants (SNVs) from twelve GWAS studies (Table S1). This set was expanded to 1,472 SNVs, referred herein as pcosSNVs, by including variants in linkage disequilibrium (LD) with GWAS-identified variants across all superpopulations (African, American, South and East Asian, European) with an r2 ≥ 0.8, obtained from SNiPA^23^. Adjacent variants were merged to define 50 genetic loci, named based on the nearest gene and/or previously associated genes in the literature (Figure 1A, Table S2). Most of these variants are located within intronic regions, with the highest density observed in the *DENND1A* and *AOPEP* loci (Figure 1A). We then assigned target genes using a recent study that predicts enhancer-gene interactions across various cell types by integrating enhancer activity, 3D chromatin interactions, and DNase I hypersensitivity maps (Methods)^24^. This analysis yielded 97 target genes (Table S3), many of which were not previously linked to PCOS. These genes are significantly enriched in pathways related to cell development, differentiation, and apoptosis (hypergeometric p-value 10^−6^, Figure 1B), highlighting their potential roles in the biological mechanisms underlying the five developmental stages of folliculogenesis in oocytes and granulosa cells^25^.

We next investigated the functional impact of variants through their association with changes in gene expression characterized by the GTEx consortium^26^. Among 1,472 pcosSNVs, 832 overlapped with cis-eQTLs, termed eVariants (Table S4). Two-thirds of these pcosSNVs, including those in the RAB5B and IRF1 loci, are shared cell-type eQTLs, meaning they influence gene expression in multiple cell types. In contrast, the remaining variants, such as those in the *FSHR* and *ERBB4* loci, are cell-type specific (Figure S1a). The number of target genes scaled almost linearly with the number of affected cell types (Spearman correlation: 0.71, Figure S1b), suggesting that shared eQTLs may contribute to distinct cell-type specific regulatory networks by regulating different genes in different cell-types. Notably, eVariants in the locus of the GATA4 gene were linked to 39 genes across 49 cell types (Figure S1b), but all were located on the same chromosome and were not detected as trans-eQTLs by GTEx, preventing confirmation of their trans-regulatory role. However, 4% of eVariants targeted long non-coding RNAs (Table S4), suggesting their role as potential trans-eQTLs^27^. Meanwhile, the remaining 640 pcosSNVs that were not identified as GTEx eVariants belong to 18 susceptibility loci, including that of *CNTNAP5*, *ASIC2*, and *CDH1*, (Table S5), likely due to low target gene expression or restricted function in specific cell states in the dynamic transcription landscape that not captured in bulk tissue analysis^26,28^. These pcosSNVs may play key spatiotemporal roles in mediating GnRH response in the hypothalamus, pituitary regulation, and follicular phase progression in PCOS^26^.

We also examined the enrichment of PCOS eVariants across GTEx cell types. Compared to a randomly selected set of 832 eQTLs (excluding PCOS eVariants), PCOS eVariants were found to be enriched in brain cell types, reproductive hormone-producing tissues such as the ovary and adrenal gland, as well as hormonally influenced tissues like the breast and prostate (p-value < 0.001, Figure 1C). Since many PCOS eVariants are shared across cell-types, they likely influence regulatory networks by mediating interactions between cell-type-specific and ubiquitous transcription factors (TFs), thereby invoking cell-type-specific regulatory pathways that contribute to distinct phenotypic outcomes in different cellular contexts^29^. For example, *PPARG*, a susceptibility locus, plays a central role in regulating lipid metabolism, adipocyte differentiation, gluconeogenesis, folliculogenesis, and steroidogenesis through multiple TFs that are critical regulators of these biological processes (www.kegg.jp/pathway/map=map03320)^30,31^. This suggests that causal variants within this locus may contribute to distinct phenotypic outcomes through pleiotropic effects across multiple cell types. Given these complexities, a deeper understanding of specific TF functions within the PCOS regulatory landscape can help to elucidate cell-type specific response in disease mechanisms.

### TFs acting as hormone response elements are enriched in pcosSNVs

Next, we explored the prevalence and importance of TFs whose binding sites are enriched for pcosSNVs. Specifically, we quantified the abundance of transcription factor binding sites (TFBSs) affected by these variants and compared it to a control set of TFBSs corresponding to 71,000 SNVs located within 100 kb of pcosSNVs (Methods). This localized background enabled us to investigate the regulation of target genes within the context of PCOS-specific biological processes, particularly for ubiquitously expressed genes. Transcription factors (TFs) whose binding sites are enriched at pcosSNVs include members of the forkhead family, notably *FOXA1*, which functions as a pioneer factor in estrogen- and androgen-mediated responses^32^, and *FOXL2*, a key ovarian transcription factor associated with primary ovarian insufficiency^33^ (p-value < 0.01, Figure 1D). Other more abundant TFBSs include that of *EGR2* that regulates ovarian cell function and survival^34,35^, as well as *WT1* and *CPEB1* that regulate granulosa cell development and proliferation^36,37^. Notably, pcosSNVs also exhibit a significant enrichment of *PROX1* binding sites—an intriguing observation given that *PROX1* itself is a known susceptibility locus for PCOS. This suggests a possible feedback regulatory mechanism involving *PROX1* in PCOS pathophysiology. We also observed enrichment of TFs selectively expressed in neuronal and ovarian cell types, such as *MNX1*, and *NKX6.1/2*, which, though not yet linked to PCOS, are interesting candidates in neuroendocrine regulation.

In parallel, we examined the transcriptional basis of differentially expressed genes (DEGs) between PCOS patients and healthy individuals in GCs—the reproductive hormone-producing cell type that governs both folliculogenesis and steroidogenesis (Methods)^38^. Using the TF enrichment pipeline from the KnockTF database, which catalogs upstream regulators of differentially expressed genes across various cell types (Methods)^39^, we identified 85 TFs whose knockdown or knockout results in differential expression of target gene sets (Figure S2, Table S6). In addition to *WT1, FOXA1*, and *EGR2*, whose binding sites are enriched in pcosSNVs, we also identified developmental regulators *SALL4* and *NANOG*, which are essential for oocyte development^40^, co-activators of estrogen and androgen signaling, including *CCAR1/2*^41,42^, and regulators of steroidogenesis, such as *AHR, NR4A1, NR5A1 (SF1)* (p-value < 0.001, Figure S2). Using the same background of 71k SNVs, we also assessed the enrichment of ChIP-Seq peaks within pcosSNVs using data from extensively profiled cell lines HepG2, HEK293, WTC11, and MCF-7 from ENCODE^24^. In HepG2, pcosSNVs were 40-fold more enriched for ChIP-Seq peaks of *CCAR2* (p-value < 10^−3^), Figure S3), which, along with *AGO2* and *RBFOX2*, was also identified as an upstream regulator of DEGs in granulosa cells (Figure S2). Interestingly, we also identified the enrichment of ChIP-Seq peaks of *NCOA2* in pcosSNVs, another coactivator of *AR* and *ESR1*. We hypothesized that some of these TFs may be involved in regulation through protein-protein interactions and not by direct binding at consensus sites^43^. Overall, this analysis highlights key regulatory TFs involved in folliculogenesis and endocrine signaling that may impact downstream pathways of circulating reproductive hormone levels in PCOS.

### PcosSNVs of reproductive and metabolic subtype are enriched for distinct TFBSs and phenotypic traits

The phenotypic co-morbidities linked to PCOS, in addition to neuroendocrine dysfunction, have led to the classification of two distinct subtypes—metabolic and reproductive. The metabolic subtype is characterized by abnormal LH:FSH ratios, SHBG levels, and/or elevated androgen levels, whereas the reproductive subtype is associated with irregular BMI, glucose, and insulin levels. Based on the sub-phenotype associations of GWAS susceptibility variants, we classified ten loci as metabolic and nineteen as reproductive (Table S7). Of note, two separate variants within the *GATA4-NEIL2* locus were linked to the reproductive and metabolic subtypes. We sought to investigate the molecular mechanisms underlying these two phenotypes.

**Figure 2:**
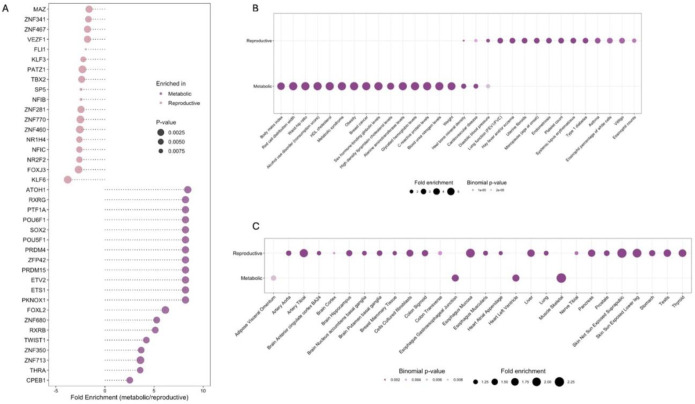
(A) Variation in TFBS enrichment between metabolic and reproductive subtypes, (B) GWAS traits associated with each subtype, (C) GTEx eQTL enrichment across the two subtypes. All reported terms meet the significance threshold of p-value < 0.01.

We first examined the differential enrichment of TFBSs in each subtype (Figure 2A, Methods). The metabolic pcosSNVs showed significant enrichment for TFBSs of *TWIST1* and *RXRB*, which regulate adipocyte differentiation^44,45^, *PTF1A*, which is essential for maintaining pancreatic cell identity^46^, and steroid hormone nuclear receptors *RXRB*, *RXRG* and *THRA*^47^ (p-value < 0.01 vs reproductive pcosSNVs). Interestingly, binding sites of TFs involved in the development and differentiation of GCs during folliculogenesis, such as *SOX2*, *CPEB1*, and *FOXL2*, are enriched in the metabolic subtype. This suggests a crucial role GCs, potentially through the regulation of reproductive hormone levels, in driving metabolic abnormalities associated with PCOS. Conversely, the reproductive subtype is enriched for TFBSs of *NR2F2*, *TBX2* and *NR1H4* (p-value < 0.01 vs metabolic pcosSNVs), all of which have well-documented roles in steroidogenesis and folliculogenesis^48,49^.

The two subtypes are enriched for variants from distinct GWAS trait groups, correlating with their phenotypic traits (Figure 2B, Methods). For example, reproductive subtype variants exhibit more than 3-fold enrichment for endocrine traits like endometriosis and uterine fibroids (p-value < 10^−13^ vs pcosSNVs), while metabolic subtype variants show significant enrichment for obesity, BMI, and cholesterol (>5-fold, p-value < 10^−30^ vs pcosSNVs). Furthermore, of the 249 metabolic and 462 reproductive variants, 203 (81%) and 253 (54%) were identified as eQTLs in GTEx cell types. Compared to metabolic eVariants, reproductive eVariants are enriched across a broader range of cell types, whereas metabolic eVariants exhibit greater cell-type specificity, particularly in the heart, adipose tissue, muscle, and esophagus (Figure 2C, Methods). The shared eQTLs among reproductive eVariants suggest potential pleiotropic effects, while the cell-type-specific eQTLs in the metabolic subtype likely exert targeted effects on their respective cell types^50^.

### Non-neuroendocrine pathogenic cell-types are proposed to manifest disease phenotype in response to impaired hormone signaling

The established role of the HPG axis in regulating circulating reproductive hormone levels highlights the hypothalamus, pituitary, adrenal gland, and ovarian granulosa and theca cells as key mediators of PCOS disease mechanisms. However, PCOS phenotypes extend beyond these cell types, affecting the pancreas, adipocytes, liver, heart, and other tissues, suggesting disruptions in downstream hormonal regulation within non-neuroendocrine systems, in addition to potential pleiotropic effects of risk variants. We thus categorized pathogenic cell-types into two groups: (a) primary pathogenic cell types, which are responsible for synthesizing reproductive hormones, and (b) secondary pathogenic cell types, which respond to these hormones through their respective receptors^51^.

**Figure 3:**
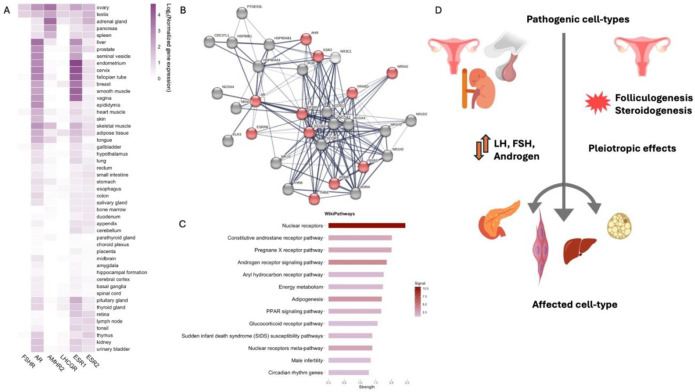
(A) Gene expression profiles of hormone receptors across cell-types obtained from the Human Protein Atlas (PubMed: 25613900), (B) Protein-protein interaction networks of TFs (in red) whose binding sites are enriched among pcosSNVs, (C) Pathways enriched in the interaction network. Nodes in red represent TFs whose binding sites are enriched in pcosSNVs. Signal is defined as a weighted harmonic mean between the observed/expected ratio and −log(FDR) and strength is Log10(observed / expected), (D) Proposed hormone mediated mechanism linking pathogenic cell-types in PCOS disease etiology.

Next, we analyzed the expression of receptors of reproductive hormones, namely, *LHCGR, FSHR, AMHR, ESR1, ESR2* and *AR*, across different cell-types (Figure 3A)^3,16,52^. We observed over 7-fold and 2-fold higher expression of *AR* in non-reproductive tissues such as the liver and muscle, respectively, compared to its mean expression across all cell types, possibly explaining the broader implications of hyperandrogenemia in PCOS patients. Additionally, *AMHR2* is also expressed in the adrenal glands but its role in the cellular context is not yet determined. Interestingly, *AR*-expressing cell types associate with traits of the metabolic subtype (Figure 2B), suggesting that co-morbidities like obesity, diabetes, and cardiovascular diseases^3^ may arise from disrupted hormone receptor networks. For instance, the role of androgens in regulating adipocyte differentiation^53^, and cholesterol biosynthesis in the liver^54^, among other functions^55^, aligns with the expression of their receptor AR across non-neuroendocrine cell-types. Additionally, the enrichment of ChIP-Seq peaks of their coactivators, such as *CCAR1/2* and *NCOA*, among pcosSNVs in HepG2 (p-value < 0.001, Figure S3) further supports the involvement of disrupted androgen signaling pathways in driving disease phenotypes in non-neuroendocrine cell types. Together, these observations indicate that understanding the regulatory networks of hormone receptors is essential to advance our understanding of the molecular mechanisms underlying PCOS as disrupted circulating hormone levels from primary pathogenic cell-types may influence other cell types through altered hormone-responsive elements, further contributing to disease pathology.

We used the set of enriched TFs in pcosSNVs and hormone receptors to obtain a protein interaction network (Figure 3B). This network is enriched for pathways related to nuclear receptors mediated hormone signaling, particularly by androgens, and adipogenesis, and metabolism (Figure 3C), highlighting a complex regulatory framework within the HPG axis governed by neuroendocrine hormones that may help in providing insights into the co-morbidities associated with PCOS. Based on these observations, we propose a signaling mechanism where dysregulated hormone biosynthesis in pathogenic cell types disrupts hormone-responsive pathways in affected cell types (Figure 3D). Additionally, pleiotropic effects of genetic variants in pathogenic cell types may influence diverse cellular processes depending on the function of the target gene (Figure 3D), explaining the multifaceted nature of PCOS.

We investigated plausible mechanisms that are disrupted in PCOS by analyzing the epigenomic features of causal variants predicted at each susceptibility locus in both pathogenic and affected cell-types using a deep learning algorithm, as described in the following section.

### Prioritizing disease causal variants using a deep learning model

The challenge of characterizing the cell-type-specific impact of thousands of susceptibility variants in complex traits and diseases has led to the development of computational approaches for inferring causal variants. DL models have been particularly effective in predicting variant effects on gene regulation by integrating diverse cell-type-specific epigenomic features^56,57^. We previously developed a convolutional neural network based DL model, TREDNet, which can predict the effects of non-coding variants on enhancer activity^58^. This two-phase DL model was demonstrated as a successful approach in prioritizing causal variants of type 2 diabetes and autism^58,59^. Building on its success, we applied TREDNet to investigate the regulatory mechanisms underlying PCOS.

**Figure 4:**
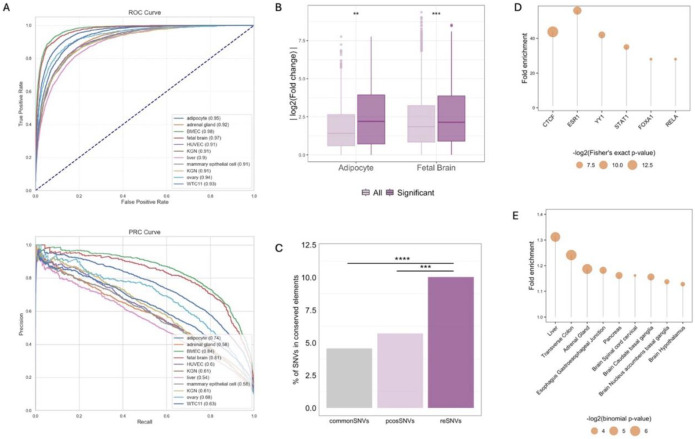
(A) ROC and PRC curves of eleven cell-type specific TREDNet models, (B) A comparison of fold change (alternate / reference allele) in TREDNet scores between all variants and those exhibiting significant change in enhancer activity in MPRA, using Wilcoxon test (ns: p > 0.05, *: p <= 0.05,**: p <= 0.01,***: p <= 0.001), (C) Fraction of SNVs overlapping with phastCons elements conserved across 30 primates, (D) TFs enriched among reSNVs exhibiting allele specific TF binding compared with pcosSNVs, (E) Fold enrichment of eVariants linked to reSNVs compared with pcosSNVs in GTEx tissues (p-value < 0.01).

We trained our model using cell-type-specific putative enhancers identified by co-occurrence of the H3K27ac histone modification with DNase hypersensitive sites (DHS), as proxies for active enhancers (Table S8, Methods). Aside from the pathogenic cell-types, we included pancreas, liver, and adipocytes to investigate regulatory changes in affected cell-types. Due to the lack of relevant epigenomic data from the pituitary and hypothalamus, we included fetal brain instead, and brain microvascular endothelial cells (BMEC), mammary epithelial cells and human umbilical vein endothelial cells (HUVEC) as proxies for granulosa cells, given their similar H3K27ac profiles (Jaccard similarity index, Table S9) and epithelial and endothelial-like properties^60^. Additionally, we incorporated WTC11, a developmental cell line, to capture causal variants active during early development, as fetal development has been implicated in PCOS onset later in life^61^. The DL models demonstrated robust performance, achieving an area under the receiver operating characteristic curve (auROC) ranging from 0.88 to 0.95 and an area under the precision-recall curve (auPRC) ranging from 0.56 to 0.85 across the seven cell types (Figure 4A).

To evaluate TREDNet’s ability to identify causal variants, we examined the correlation between TREDNet-predicted differences in allele-specific enhancer activity and those determined through a massively parallel reporter assay (MPRA) in the developing human brain and stem cell-derived adipocytes using our model trained on fetal brain and adipocytes^62,63^. We compared allele-specific TREDNet scores across all assayed alleles and those showing significant changes in reporter activity (Methods) and observed a significantly higher fold change in TREDNet scores for the latter group (Mann-Whitney test p-value = 0.001 for adipocytes and 10^−5^ for fetal brain, Figure 4B). These findings highlight TREDNet’s robustness in predicting causal variants across different cell types.

Next, we evaluated the impact of pcosSNVs within active regulatory regions across the eleven selected cell-types, scoring both reference and risk alleles in all cell-type-specific models (Methods). For pcosSNVs located in active regulatory regions marked by H3K4me1, H3K27ac, or DNase/ATAC-Seq, we classified strengthening alleles as those with scores below the threshold (determined at 10% FDR) for the reference allele and above for the risk allele, while damaging alleles followed the opposite criterion. Applying this approach, we identified 309 pcosSNVs with predicted allelic differences in activity, termed reSNVs (Table S10). These reSNVs were significantly enriched in conserved elements compared to both pcosSNVs and 13 million common SNVs from the 1000 Genomes catalog (binomial p-value = 0.0002 and 10^−9^, respectively, Figure 4C). Moreover, reSNVs exhibited greater enrichment in the brain, liver, adrenal gland, and pancreas compared to pcosSNVs (Figure 4D), suggesting these as key cell-types impacted by reSNVs.

To further assess the functional impact of reSNVs, we examined their influence on transcription factor (TF) binding using allele-specific binding (ASB) data for ~400 TFs across 557 cell types from the ADASTRA database (Methods)^64^. reSNVs showed a four-fold enrichment in ASB SNVs compared to pcosSNVs (binomial p-value < 10^−24^). Notably, significantly affected TF binding sites included those for *FOXA1* and *ESR1* (Figure 4E), suggesting the regulatory impact of reSNVs through hormone responsive elements.

Our deep learning-based approach identified 20% of the pcosSNVs as potential regulatory variants, effectively narrowing down causal variants in loci such as *ERBB4*, *LHCGR, MC4R*, etc (Figure S4). For example, among 51 variants in the *ERBB4* locus, we identified rs79230362 as an enhancer-disrupting variant in HUVEC cells (Figure S5). This variant is in LD with the GWAS SNP rs113168128 and is predicted to disrupt the binding site of the *ELK1:SREBF2* motif complex (Figure S5). Given the established role of *SREBF2* in steroidogenesis^65^ and the highly cell-type-specific expression of ELK1 in granulosa cells (Figure S5), this variant likely affects *ERBB4* expression, a key regulator of the oocyte microenvironment during folliculogenesis^18^. Similarly, in the *MC4R* locus, we identified rs17773430 as a causal enhancer-disrupting variant in WTC11 cells. *MC4R* is a critical component of the melanocortin pathway and a well-established obesity susceptibility gene that is also linked to PCOS. Notably, knockout studies of *MC4R* in mice result in both obesity and infertility phenotypes, highlighting shared regulatory architectures underlying these conditions^66^. rs17773430 is predicted to disrupt the binding site of *TBX2/TBXT*, TFs responsible for the development of hypothalamus-pituitary axis^48^. Given that reduced *MC4R* levels are associated with lower LH levels^67^, this variant likely contributes to PCOS etiology through its impact on HPG axis.

On the other hand, multiple reSNVs were identified in the locus of *DENND1A*, *FTO* and *MAPRE1* (Figure S6). The significant overlap of reSNVs in *DENND1A* and *MAPRE1* locus with active regulatory regions in the fetal brain and WTC11 suggests their potential role in disease manifestation during early development. Notably, a reSNV in the *MAPRE1* locus, rs187178, was validated as an enhancer-disrupting variant in the fetal brain and functions as an eQTL for the neighboring gene *DNMT3B*, which regulates dynamic methylation transitions during folliculogenesis^25^. In total, we identified 12 reSNVs validated as enhancer disrupting variants in adipocytes and fetal brain through MPRA studies (Table S11)^62,63^.

Additionally, we gained insights into the potential role of certain loci whose involvement in PCOS etiology is currently uncharacterized. For example, we identified a reSNV, rs1784692, in the *ZBTB16* locus, which exhibited the highest predicted enhancer-strengthening potential in the fetal brain, pancreas, adipocytes, WTC11, and liver (Figure S7A). The T→C polymorphism enhances *AR* receptor binding (Figure S7B), suggesting a possible association of this locus with cell-type-specific androgen response functions, such as insulin secretion in the pancreas^68^, and regulation of adipocyte differentiation^45^. While *ZBTB16*’s role in PCOS remains unexplored, its protein interaction network is enriched for androgen signaling (Figure S7C). These findings suggest that *ZBTB16* may function as a susceptibility locus involved in androgen-mediated responses that are disrupted in PCOS.

Of note, epigenomic data from fetal brain used by the DL model failed to capture the regulatory impact of pathogenic variants in the *FSHB* locus, including rs10835638 and rs11031006, which have been experimentally shown to reduce *FSHB* expression restricted to the pituitary gland^69^. This underscores the necessity of incorporating additional, relevant cell types for a more comprehensive study of the regulatory landscape of PCOS, when experimental characterization of chromatin marks becomes available for these cell types.

### The FTO locus demonstrates disruption of an androgen mediated network pleiotropy

The regulatory locus within the intronic region of *FTO* is a well-known susceptibility locus with significant implications in obesity and diabetes. Notably, it has been experimentally validated to function as a distal enhancer of *IRX3*, a TF in PCOS-associated susceptibility loci^70,71^. We hypothesized that this locus may have broader pleiotropic effects across different cell types due to variations in the expression of *IRX3*, which may influence multiple biological pathways^72^. Interestingly, the PCOS susceptibility variants localize in the genomic region regulating *IRX3* (chr16:53731249–54975288)^73^, suggesting that *IRX3* is likely the target gene of the PCOS susceptibility locus as well (Figure S8).

**Figure 5:**
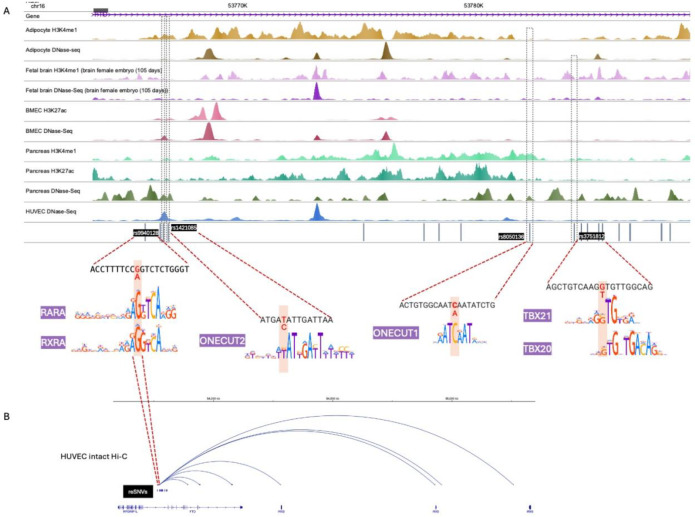
reSNVs in FTO locus exhibiting significant fold change in TREDNet predicted enhancer activity. (A) Overlap of reSNVs with active regulatory regions of pathogenic cell-types (B) Intact Hi-C map of chromatin interactions from reSNVs in FTO locus in HUVEC (doi:10.17989/ENCSR788FBI)

We identified 12 reSNVs exhibiting significant fold changes across five cell types (Figure S9). Among these, three variants— rs1421085, rs9940646 and rs9940128—have been validated by MPRA studies to show allelic changes in enhancer activity in mouse preadipocyte and neuronal cell lines^71^, further supporting the predictive accuracy of TREDNet in identifying causal variants. Interestingly, we predicted that rs1421085 additionally upregulates enhancer activity in BMEC by potentially modulating the binding site of *ONECUT2* (Figure 5A), a suppressor of androgen receptor signaling which was recently identified as a marker of follicle growth^74,75^.

Additionally, we identified another variant within the same locus, rs8050136, which is predicted as a causal variant in the pancreas and liver (Figure 5A, S8). This variant functions as an eQTL for *IRX3* in the pancreas, where IRX3 regulates the conversion of beta to epsilon cells, directly linking it to type 2 diabetes^76^. Notably, rs8050136 is also predicted to disrupt the binding site for *ONECUT1*, a transcription factor critical for pancreatic development (Figure 5). Together, these findings suggest that rs8050136 may serve as another causal variant for type 2 diabetes, possibly preferentially in PCOS patients.

To address the association of these variants with PCOS, we focused on a previous study that identified *IRX3* and the another gene in this susceptibility locus, *IRX5*, as key regulators of folliculogenesis in granulosa cells^77^. Using evidence from granulosa like cells, BMEC and HUVEC, we hypothesize that variants in this locus lead to impaired folliculogenesis, consequently disrupting androgen production in the pathogenic cell type—likely granulosa cells—through the dysregulated action of *IRX3/IRX5*. This disruption in androgen production may have pleiotropic effects on other cell types where these genes function within the androgen-responsive network, aligning with our proposed mechanism (Figure 3D). In this regard, rs9940128 emerges as a plausible causal variant as it forms chromatin contacts with promoters of *IRX3* and *IRX5* (Figure 5B) and is predicted to cause a significant fold change in enhancer activity in BMEC and HUVEC (Figure S9). Furthermore, the allelic effects of variants in this locus may also impact *IRX3/5*-mediated functions in hypothalamic neurons (Figure S8), as demonstrated in mice^71^. To explore this further, we analyzed the impact of these variants in fetal brain and found that rs3751812 is located within binding sites of T-box family TFs (Figure 5). Notably, members of the T-box family play a critical role in the commitment of hypothalamus and pituitary lineages from neuronal precursors^48,78^. However, given the short temporal window of expression of these TFs in neuronal development, inferring causal mechanisms remains challenging. This highlights the necessity of using epigenomic datasets across different developmental timepoints for a comprehensive investigation.

The potential pleiotropic impact of disease-associated variants in non-pathogenic cell types is often buffered by robust regulatory networks, preventing overt disease manifestation. This suggests that assessing polygenic risk scores may be necessary to fully understand their contribution to disease susceptibility. Given that variants in the *FTO* locus have high minor allele frequencies (>0.4), which far exceed the prevalence of PCOS, it is evident that the disease phenotypes emerge from the cumulative effects of multiple dysregulated genes and pathways. Further investigations into polygenic interactions and gene-environment influences will be essential to expand our understanding of the complexity of PCOS.

## Discussion

Our limited understanding of the regulatory landscape of PCOS stems from its complex genetic architecture, which presents with heterogeneous phenotypes across different cell types, individuals, and populations. This complexity has necessitated evolving diagnostic criteria as our knowledge of the underlying pathophysiology expands. Several key questions remain unresolved, including the genetic and molecular origins of reproductive and metabolic dysfunction, the role of androgens and other hormones in regulatory pathways, and the inheritance patterns affecting both males and females. To date, GWAS have identified 50 genomic loci associated with PCOS across diverse populations (Table S2). While the functional significance of genes such as *ERBB4*, *PPARG*, and *IRX3* has been well established—leading to the use of their agonists as potential treatments^18,31,77^ —the precise molecular mechanisms remain elusive. Additionally, advancements in whole-genome and exome sequencing continue to uncover novel loci, further complicating our understanding of PCOS and highlighting the need for a deeper exploration of the core regulatory mechanisms driving its pathophysiology.

Leveraging extensive genetic and epigenetic data, we sought to identify key mechanisms linking PCOS susceptibility loci. We found that the susceptibility loci are enriched for TFBSs associated with folliculogenesis, including *WT1*, *CPEB1*, and *FOXL1*. Subtype-specific enrichment of distinct TFs provided deeper insights into the underlying biological pathways. TFs such as *PTF1A*, *RXRB/G*, and *TWIST1*, which are involved in adipocyte and pancreatic differentiation, were enriched in the metabolic subtype, while steroidogenesis- and folliculogenesis-related TFs, including *NR2F2* and *NR1H4*, were enriched in the reproductive subtype. By integrating these findings with an *in-silico* causal variant assessment, we propose that neuroendocrine abnormalities in PCOS arise from disruptions in hormone biosynthesis and/or their release from pathogenic cell-types, manifesting as distinct phenotypes through regulatory interactions of their receptors in affected cell-types. By prioritizing variants that disrupt PCOS associated TF binding sites at susceptibility loci, we highlight the importance of TFs interacting with hormone receptors—particularly androgens—as key modulators of PCOS-related dysfunction. Our results highlight the need for further characterization of TFs such as the *TBX* family, *MNX1*, etc., along with their interactions with hormonal receptors, to gain deeper insights into PCOS regulatory biology.

Our prioritization of multiple variants at each susceptibility locus is based on the one-gene multiple enhancers common assumption, according to which, the expression of a target gene can be influenced by more than one variant^71,79^. For example, two distinct variants in the *FSHB* locus—rs10835638 and rs11031006—alter *FSHB* expression, ultimately contributing to infertility^69^. These variants may occur in different individuals, leading to distinct, individual-specific phenotypes depending on the cell-type-specific networks they modulate in a pleiotropic manner. However, a common theme among reSNVs is their enrichment in reproductive hormone-responsive elements, such as *FOXA1* and *ESR1* (Figure 4D), along with their interacting transcription factors, including *ONECUT1/2*. This suggests that impaired downstream androgen signaling may be a key mechanism contributing to PCOS pathophysiologys

The susceptibility loci of PCOS implicate genes such as *ZBTB16*, *AOPEP*, *THADA*, and *CCDC91* (Figure 1A), which are ubiquitously expressed, raising the question of how disease-specific variants selectively affect certain cell types. At the molecular level, follicle progression involves signaling pathways like TGFβ, Hippo, Wnt, and mTOR, which regulate fundamental processes such as cell proliferation, differentiation, and apoptosis^7^. Why, then, do complex diseases manifest in only a subset of susceptible cell types? In the case of *ZBTB16*, we predicted that rs1784692 strengthens enhancer activity by increasing the binding affinity of *AR*, thereby implicating *ZBTB16* in downstream pathways of androgen signaling. This suggests that perturbations in disease-relevant TF interactions, specific to causal cell types, disrupt molecular networks in a way that surpasses compensatory mechanisms in other cell types, thereby making certain cells uniquely vulnerable. Consequently, transcription factors act as primary responders to disease-associated alterations, preceding the genes they regulate, and may therefore serve as more informative markers of disease susceptibility than the genes themselves.

Our analysis of the PCOS regulatory landscape reveals unifying molecular mechanisms underlying disease phenotypes. However, a more comprehensive understanding of gene regulatory networks requires integrating epigenomic datasets from key pathogenic cell types—such as the pituitary gland, granulosa, and theca cells, and potentially, the hypothalamus—across follicular phases to map the spatiotemporal regulation of genes involved in steroidogenesis and folliculogenesis. Despite the hypothalamus’s central role in the HPG axis, regulatory networks mediated by GnRH signaling remain poorly understood. Disruptions in this pathway may explain the involvement of risk genes such as *CNTNAP5*, *ASIC2*, and *CUX2*, potentially linking PCOS to prevalent mental health disorders^3^. Incorporating these datasets can enable the development of more inclusive deep-learning models capable of predicting regulatory activity changes beyond enhancer disruptions, offering deeper insights into PCOS pathophysiology.

Additionally, our PCOS subtype classification remains incomplete due to lack of data, leaving some loci unassigned, which may exclude crucial transcription factors and interactions essential for understanding regulatory networks. Lastly, our analysis of causal variants was limited to those occurring within putative enhancers. However, variants can impact gene regulation beyond enhancer activity. Variants located in silencers or insulators may disrupt distal enhancer interactions, as observed with IRX3, emphasizing the need for Hi-C data from pathogenic and affected cell types to resolve target genes not identifiable through eQTL analysis. Lastly, a comprehensive approach should also consider the trans-regulatory effects of risk variants—whether through TFs encoded by susceptibility loci (*PROX1*, *SOX5/8*, *IRF1*) or non-coding RNAs that contribute to epigenomic regulation of gene expression.

While preliminary, our results provide valuable insights into molecular mechanisms underlying PCOS etiology. Future *in vitro* and *in vivo* characterization will be essential to validate these predictions, potentially paving the way for novel, symptom-targeted therapies for PCOS patients.

## Methods

### PCOS susceptibility loci

PCOS GWAS summary statistics were obtained from the NHGRI-GWAS catalog. Variants in LD were expanded and clustered into 50 loci based on 100kb proximity. Loci subtypes were assigned using GWAS-mapped traits and supporting literature (Table S7), then translated to LD variants. The risk allele served as the alternate allele for GWAS variants, while the minor allele was used for LD variants. All analyses were conducted using the GRCh38 reference genome.

### Transcription factor binding sites

Transcription factor binding site (TFBS) regions were defined by extending variant sites by 30 bp on each side. TF binding profiles from HOCOMOCO^80^ and JASPAR non redundant collection^81^ were analyzed using FIMO with default parameters^82^. Aside from gain and loss of motifs, changes in motif scores were used to assess affinity differences between reference and alternate alleles. A list of all the TFBSs gained, lost and modulated for SNPs exhibiting significant fold change is provided in Table S10.

Differential enrichment of TFBSs between the metabolic and reproductive subtypes was assessed using a binomial test, with TFBSs overlapping variants of one subtype analyzed against the TFBSs of the other subtype as the background.

### Cell type specific DL models

We used a two phase TREDNet model developed in our lab for cell-type specific enhancer prediction^83^. The first phase of the model was pre-trained on 4560 genomic and epigenomic profiles, which included DHS, ATAC-Seq, Histone ChIP-Seq and and TF ChIP-Seq peaks from ENCODE v4^84^. The second phase was fine-tuned to predict cell type specific enhancers using training datasets described below. Chromosomes 8 and 9 were held out for testing, chromosome 6 was used for validation and other autosomal chromosomes were used to build the second phase model. The area under the ROC and PRC curve for each of these models is listed in Table S9. The pre-trained phase-one model has been deposited at https://doi.org/10.5281/zenodo.8161621.

Open chromatin (DHS or ATAC-Seq) and H3K27ac profiles for the causal cell-types were downloaded from ENCODE^84^ (Table S6). Positive datasets were defined as 2 kb regions centered on overlaps between H3K27ac ChIP-seq peaks and chromatin accessibility peaks of each cell type, excluding coding sequences, promoter proximal regions (<2kb from TSS) and ENCODE blacklisted regions^85^. A 10-fold control dataset was generated for each cell-type using randomly sampled 2kb fragments of the genome, excluding the positive dataset of that cell type and blacklisted regions.

Each 2 kb fragment received an enhancer probability score. Active enhancers were predicted at a 10% FPR with a 1:10 positive-to-control ratio. Variant effects were assessed by scoring 2 kb regions centered on each variant for reference and alternate alleles. A significant enhancer activity change was defined as an alternate/reference score ratio >1.2 or <0.8.

### Enrichment analysis of TFBSs

We used command line FIMO^86^ to scan vertebrate TF motifs from JASPAR^87^ and HOCOMOCO^88^ databases along the sequences, applying a p-value threshold of 10^−5^. Enrichment analysis used a background set of 1000 Genomes variants located within 100 kb of pcosSNVs but excluding those overlapping 30 bp flanks of pcosSNVs, exons and promoter regions (UCSC-defined). Enrichment analysis was restricted to those TFBSs with at least 10% occurrence relative to controls. Differential TFBS abundance in reproductive vs. metabolic subtype variants was determined using the ratio of normalized TF binding site counts between these enhancer categories.

### Data and tools

The H3K27ac peaks for KGN cells and adipocytes were sourced from literature^89,90^. The KGN wig file was converted to NarrowPeak format using UCSC BigWig tools^91^ and MACS peak calling software^92^.

Motif logos were retrieved from HOCOMOCO database^88^. Ontology enrichment of pcosSNVs was performed using the Molecular Signatures Database^93^. Protein interaction networks and enriched pathways (Figure 3C) were obtained from STRING database^94^. Evolutionary conservation of genomic regions was measured by their extent of overlap with phastCons elements conserved across 30 primates (https://hgdownload.soe.ucsc.edu/goldenPath/hg38/database/phastConsElements30way.txt.gz).

## Supplementary Material

Supplement 1

Supplement 2

## Data Availability

Please see the section “Data and tools” and supplementary tables.

## References

[1] Joham A. E. Polycystic ovary syndrome. Lancet Diabetes Endocrinol. 10, 668–680 (2022). 35934017 10.1016/S2213-8587(22)00163-2

[2] Palomba S., Piltonen T. T. & Giudice L. C. Endometrial function in women with polycystic ovary syndrome: a comprehensive review. Hum. Reprod. Update 27, 584–618 (2021). 33302299 10.1093/humupd/dmaa051

[3] Stener-Victorin E. Polycystic ovary syndrome. Nat. Rev. Dis. Primer 10, 1–23 (2024). 10.1038/s41572-024-00511-3 38637590

[4] Zaidi M., Yuen T. & Kim S.-M. Pituitary crosstalk with bone, adipose tissue and brain. Nat. Rev.Endocrinol. 19, 708–721 (2023). 37715028 10.1038/s41574-023-00894-5 PMC11730177

[5] McCartney C. R. & Marshall J. C. Polycystic Ovary Syndrome. N. Engl. J. Med. 375, 54–64 (2016). 27705264 10.1056/NEJMc1610000

[6] Abedel-Majed M. A., Romereim S. M., Davis J. S. & Cupp A. S. Perturbations in Lineage Specification of Granulosa and Theca Cells May Alter Corpus Luteum Formation and Function. Front. Endocrinol. 10, (2019). 10.3389/fendo.2019.00832 PMC6895843 31849844

[7] Wang K. & Li Y. Signaling pathways and targeted therapeutic strategies for polycystic ovary syndrome. Front. Endocrinol. 14, 1191759 (2023). 10.3389/fendo.2023.1191759 PMC10622806 37929034

[8] Liao B., Qiao J. & Pang Y. Central Regulation of PCOS: Abnormal Neuronal-Reproductive Metabolic Circuits in PCOS Pathophysiology. Front. Endocrinol. 12, 667422 (2021). 10.3389/fendo.2021.667422 PMC8194358 34122341

[9] Zhang C.-H., Liu X.-Y. & Wang J. Essential Role of Granulosa Cell Glucose and Lipid Metabolism on Oocytes and the Potential Metabolic Imbalance in Polycystic Ovary Syndrome. Int. J. Mol. Sci. 24,16247 (2023). 38003436 10.3390/ijms242216247 PMC10671516

[10] Dapas M. & Dunaif A. Deconstructing a Syndrome: Genomic Insights Into PCOS Causal Mechanisms and Classification. Endocr. Rev. 43, 927–965 (2022). 35026001 10.1210/endrev/bnac001 PMC9695127

[11] Gorsic L. K., Dapas M., Legro R. S., Hayes M. G. & Urbanek M. Functional Genetic Variation in the Anti-Müllerian Hormone Pathway in Women With Polycystic Ovary Syndrome. J. Clin.Endocrinol. Metab. 104, 2855–2874 (2019). 30786001 10.1210/jc.2018-02178 PMC6543512

[12] Caburet S. A homozygous mutation of GNRHR in a familial case diagnosed with polycystic ovary syndrome. Eur. J. Endocrinol. 176, K9–K14 (2017). 28348023 10.1530/EJE-16-0968

[13] Chen Y. Genetic and Epigenetic Landscape for Drug Development in Polycystic Ovary Syndrome. Endocr. Rev. 45, 437–459 (2024). 38298137 10.1210/endrev/bnae002

[14] Vink J. M., Sadrzadeh S., Lambalk C. B. & Boomsma D. I. Heritability of polycystic ovary syndrome in a Dutch twin-family study. J. Clin. Endocrinol. Metab. 91, 2100–2104 (2006). 16219714 10.1210/jc.2005-1494

[15] Wang F. Alternative splicing of the androgen receptor in polycystic ovary syndrome. Proc. Natl. Acad. Sci. U. S. A. 112, 4743–4748 (2015). 25825716 10.1073/pnas.1418216112 PMC4403157

[16] Muccee F. Exploring the association of ESR1 and ESR2 gene SNPs with polycystic ovary syndrome in human females: a comprehensive association study. J. Ovarian Res. 17, 27 (2024). 38281964 10.1186/s13048-023-01335-7 PMC10823698

[17] Mathieson I. The omnigenic model and polygenic prediction of complex traits. Am. J. Hum. Genet. 108, 1558–1563 (2021). 34331855 10.1016/j.ajhg.2021.07.003 PMC8456163

[18] Veikkolainen V. Erbb4 regulates the oocyte microenvironment during folliculogenesis. Hum.Mol. Genet. 29, 2813–2830 (2020). 32716031 10.1093/hmg/ddaa161

[19] LaVoie H. A. The GATA-keepers of ovarian development and folliculogenesis. Biol. Reprod. 91, 38 (2014). 24990805 10.1095/biolreprod.114.122499

[20] Waterbury J. S. The PCOS GWAS Candidate Gene ZNF217 Influences Theca Cell Expression of DENND1A.V2, CYP17A1, and Androgen Production. J. Endocr. Soc. 6, bvac078 (2022). 35668995 10.1210/jendso/bvac078 PMC9155636

[21] Li M. The HMGA2-IMP2 Pathway Promotes Granulosa Cell Proliferation in Polycystic Ovary Syndrome. J. Clin. Endocrinol. Metab. 104, 1049–1059 (2019). 30247605 10.1210/jc.2018-00544 PMC6753588

[22] Xi Y. HMGA2 promotes adipogenesis by activating C/EBPβ-mediated expression of PPARγ. Biochem. Biophys. Res. Commun. 472, 617–623 (2016). 26966068 10.1016/j.bbrc.2016.03.015

[23] Arnold M., Raffler J., Pfeufer A., Suhre K. & Kastenmüller G. SNiPA: an interactive, genetic variant-centered annotation browser. Bioinforma. Oxf. Engl. 31, 1334–1336 (2015). 10.1093/bioinformatics/btu779 PMC4393511 25431330

[24] Gschwind A. R. An encyclopedia of enhancer-gene regulatory interactions in the human genome. bioRxiv 2023.11.09.563812 (2023) doi:10.1101/2023.11.09.563812.

[25] Zhang Y. Transcriptome Landscape of Human Folliculogenesis Reveals Oocyte and Granulosa Cell Interactions. Mol. Cell 72, 1021–1034.e4 (2018). 30472193 10.1016/j.molcel.2018.10.029

[26] Consortium GTEx Genetic effects on gene expression across human tissues. Nature 550, 204–213 (2017). 29022597 10.1038/nature24277 PMC5776756

[27] Bai Y., Dai X., Harrison A. P. & Chen M. RNA regulatory networks in animals and plants: a long noncoding RNA perspective. Brief. Funct. Genomics 14, 91–101 (2015). 24914100 10.1093/bfgp/elu017

[28] Fairfax B. P. Genetics of gene expression in primary immune cells identifies cell type-specific master regulators and roles of HLA alleles. Nat. Genet. 44, 502–510 (2012). 22446964 10.1038/ng.2205 PMC3437404

[29] Sonawane A. R. Understanding Tissue-Specific Gene Regulation. Cell Rep. 21, 1077–1088 (2017). 29069589 10.1016/j.celrep.2017.10.001 PMC5828531

[30] Han L., Shen W.-J., Bittner S., Kraemer F. B. & Azhar S. PPARs: regulators of metabolism and as therapeutic targets in cardiovascular disease. Part II: PPAR-β/δ and PPAR-γ. Future Cardiol. 13, 279–296 (2017). 28581362 10.2217/fca-2017-0019 PMC5941699

[31] Kim J., Bagchi I. C. & Bagchi M. K. Control of ovulation in mice by progesterone receptor-regulated gene networks. Mol. Hum. Reprod. 15, 821–828 (2009). 19815644 10.1093/molehr/gap082 PMC2776476

[32] Lupien M. FoxA1 translates epigenetic signatures into enhancer-driven lineage-specific transcription. Cell 132, 958–970 (2008). 18358809 10.1016/j.cell.2008.01.018 PMC2323438

[33] Georges A. FOXL2: a central transcription factor of the ovary. J. Mol. Endocrinol. 52, R17–33 (2014). 24049064 10.1530/JME-13-0159

[34] Lee S. L. Luteinizing hormone deficiency and female infertility in mice lacking the transcription factor NGFI-A (Egr-1). Science 273, 1219–1221 (1996). 8703054 10.1126/science.273.5279.1219

[35] Jin H. EGR2 is a gonadotropin-induced survival factor that controls the expression of IER3 in ovarian granulosa cells. Biochem. Biophys. Res. Commun. 482, 877–882 (2017). 27890615 10.1016/j.bbrc.2016.11.127

[36] Cen C. Inactivation of Wt1 causes pre-granulosa cell to steroidogenic cell transformation and defect of ovary development†. Biol. Reprod. 103, 60–69 (2020). 32301970 10.1093/biolre/ioaa042

[37] Xuan F. CPEB1 induces autophagy and promotes apoptosis in ovarian granulosa cells of polycystic ovary syndrome. Mol. Reprod. Dev. 91, e23741 (2024). 38616716 10.1002/mrd.23741

[38] Li J. Molecular Features of Polycystic Ovary Syndrome Revealed by Transcriptome Analysis of Oocytes and Cumulus Cells. Front. Cell Dev. Biol. 9, 735684 (2021). 34552933 10.3389/fcell.2021.735684 PMC8450412

[39] Feng C. KnockTF 2.0: a comprehensive gene expression profile database with knockdown/knockout of transcription (co-)factors in multiple species. Nucleic Acids Res. 52, D183–D193 (2024). 37956336 10.1093/nar/gkad1016 PMC10767813

[40] Overland M. R. Development of the human ovary: Fetal through pubertal ovarian morphology, folliculogenesis and expression of cellular differentiation markers. Differ. Res. Biol.Divers. 129, 37–59 (2023). 10.1016/j.diff.2022.10.005 36347737

[41] Trauernicht A. M., Kim S. J., Kim N. H. & Boyer T. G. Modulation of estrogen receptor alpha protein level and survival function by DBC-1. Mol. Endocrinol. Baltim. Md 21, 1526–1536 (2007). 10.1210/me.2007-0064 17473282

[42] Fu J. Deleted in breast cancer 1, a novel androgen receptor (AR) coactivator that promotes AR DNA-binding activity. J. Biol. Chem. 284, 6832–6840 (2009). 19126541 10.1074/jbc.M808988200 PMC2652261

[43] Jindal G. A. & Farley E. K. Enhancer grammar in development, evolution, and disease: dependencies and interplay. Dev. Cell 56, 575–587 (2021). 33689769 10.1016/j.devcel.2021.02.016 PMC8462829

[44] Siersbæk R. Dynamic Rewiring of Promoter-Anchored Chromatin Loops during Adipocyte Differentiation. Mol. Cell 66, 420–435.e5 (2017). 28475875 10.1016/j.molcel.2017.04.010

[45] Sun L. Deciphering the interaction between Twist1 and PPARγ during adipocyte differentiation. Cell Death Dis. 14, 764 (2023). 37996425 10.1038/s41419-023-06283-0 PMC10667345

[46] Weedon M. N. Recessive mutations in a distal PTF1A enhancer cause isolated pancreatic agenesis. Nat. Genet. 46, 61–64 (2014). 24212882 10.1038/ng.2826 PMC4131753

[47] Evans R. M. & Mangelsdorf D. J. Nuclear Receptors, RXR, and the Big Bang. Cell 157, 255–266 (2014). 24679540 10.1016/j.cell.2014.03.012 PMC4029515

[48] Pontecorvi M., Goding C. R., Richardson W. D. & Kessaris N. Expression of Tbx2 and Tbx3 in the developing hypothalamic-pituitary axis. Gene Expr. Patterns GEP 8, 411–417 (2008). 18534921 10.1016/j.gep.2008.04.006

[49] Candelaria N. R. & Richards J. S. Targeted deletion of NR2F2 and VCAM1 in theca cells impacts ovarian follicular development: insights into polycystic ovary syndrome?†. Biol. Reprod. 110, 782–797 (2024). 38224314 10.1093/biolre/ioae010 PMC11017119

[50] Gamazon E. R. Using an atlas of gene regulation across 44 human tissues to inform complex disease- and trait-associated variation. Nat. Genet. 50, 956–967 (2018). 29955180 10.1038/s41588-018-0154-4 PMC6248311

[51] Concannon P., Rich S. S. & Nepom G. T. Genetics of type 1A diabetes. N. Engl. J. Med. 360, 1646–1654 (2009). 19369670 10.1056/NEJMra0808284

[52] Wu P.-F. Polycystic ovary syndrome is causally associated with estrogen receptor-positive instead of estrogen receptor-negative breast cancer: a Mendelian randomization study. Am. J. Obstet. Gynecol. 223, 583–585 (2020). 32413428 10.1016/j.ajog.2020.05.016

[53] Chazenbalk G. Androgens inhibit adipogenesis during human adipose stem cell commitment to preadipocyte formation. Steroids 78, 920–926 (2013). 23707571 10.1016/j.steroids.2013.05.001 PMC3951890

[54] Krycer J. R. & Brown A. J. Cross-talk between the androgen receptor and the liver X receptor: implications for cholesterol homeostasis. J. Biol. Chem. 286, 20637–20647 (2011). 21489984 10.1074/jbc.M111.227082 PMC3121513

[55] Davey R. A. & Grossmann M. Androgen Receptor Structure, Function and Biology: From Bench to Bedside. Clin. Biochem. Rev. 37, 3–15 (2016). 27057074 PMC4810760

[56] Zhou J. Deep learning sequence-based ab initio prediction of variant effects on expression and disease risk. Nat. Genet. 50, 1171–1179 (2018). 30013180 10.1038/s41588-018-0160-6 PMC6094955

[57] Avsec Ž. Effective gene expression prediction from sequence by integrating long-range interactions. Nat. Methods 18, 1196–1203 (2021). 34608324 10.1038/s41592-021-01252-x PMC8490152

[58] Hudaiberdiev S. Modeling islet enhancers using deep learning identifies candidate causal variants at loci associated with T2D and glycemic traits. Proc. Natl. Acad. Sci. U. S. A. 120, e2206612120 (2023). 37603758 10.1073/pnas.2206612120 PMC10469333

[59] Li S., Hannenhalli S. & Ovcharenko I. De novo human brain enhancers created by single-nucleotide mutations. Sci. Adv. 9, eadd2911 (2023). 36791193 10.1126/sciadv.add2911 PMC9931207

[60] Antczak M. & Van Blerkom J. The vascular character of ovarian follicular granulosa cells: phenotypic and functional evidence for an endothelial-like cell population. Hum. Reprod. Oxf. Engl. 15, 2306–2318 (2000). 10.1093/humrep/15.11.2306 11056124

[61] Hartanti M. D. Could perturbed fetal development of the ovary contribute to the development of polycystic ovary syndrome in later life? PloS One 15, e0229351 (2020). 32078641 10.1371/journal.pone.0229351 PMC7032716

[62] Joslin A. C. A functional genomics pipeline identifies pleiotropy and cross-tissue effects within obesity-associated GWAS loci. Nat. Commun. 12, 5253 (2021). 34489471 10.1038/s41467-021-25614-3 PMC8421397

[63] Deng C. Massively parallel characterization of regulatory elements in the developing human cortex. Science 384, eadh0559 (2024). 38781390 10.1126/science.adh0559 PMC12085231

[64] Abramov S. Landscape of allele-specific transcription factor binding in the human genome. Nat. Commun. 12, 2751 (2021). 33980847 10.1038/s41467-021-23007-0 PMC8115691

[65] Nakanishi T. LH Induces De Novo Cholesterol Biosynthesis via SREBP Activation in Granulosa Cells During Ovulation in Female Mice. Endocrinology 162, bqab166 (2021). 34431998 10.1210/endocr/bqab166

[66] Talbi R. POMC neurons control fertility through differential signaling of MC4R in Kisspeptin neurons. BioRxiv Prepr. Serv. Biol. 2024.02.18.580873 (2024) doi:10.1101/2024.02.18.580873. PMC12270483 40674128

[67] Villa P. A., Ruggiero-Ruff R. E., Jamieson B. B., Campbell R. E. & Coss D. Obesity Alters POMC and Kisspeptin Neuron Cross Talk Leading to Reduced Luteinizing Hormone in Male Mice. J. Neurosci. Off. J. Soc. Neurosci. 44, e0222242024 (2024). 10.1523/JNEUROSCI.0222-24.2024 PMC11236585 38744532

[68] Karagiannopoulos A. Glucocorticoid-mediated induction of ZBTB16 affects insulin secretion in human islets and EndoC-βH1 β-cells. iScience 26, 106555 (2023). 37250333 10.1016/j.isci.2023.106555 PMC10214295

[69] Bohaczuk S. C., Thackray V. G., Shen J., Skowronska-Krawczyk D. & Mellon P. L. FSHB Transcription is Regulated by a Novel 5’ Distal Enhancer With a Fertility-Associated Single Nucleotide Polymorphism. Endocrinology 162, bqaa181 (2021). 33009549 10.1210/endocr/bqaa181 PMC7846141

[70] Smemo S. Obesity-associated variants within FTO form long-range functional connections with IRX3. Nature 507, 371–375 (2014). 24646999 10.1038/nature13138 PMC4113484

[71] Sobreira D. R. Extensive pleiotropism and allelic heterogeneity mediate metabolic effects of IRX3 and IRX5. Science 372, 1085–1091 (2021). 34083488 10.1126/science.abf1008 PMC8386003

[72] Brynedal B. Large-Scale trans-eQTLs Affect Hundreds of Transcripts and Mediate Patterns of Transcriptional Co-regulation. Am. J. Hum. Genet. 100, 581–591 (2017). 28285767 10.1016/j.ajhg.2017.02.004 PMC5384037

[73] Claussnitzer M. FTO Obesity Variant Circuitry and Adipocyte Browning in Humans. N. Engl. J.Med. 373, 895–907 (2015). 26287746 10.1056/NEJMoa1502214 PMC4959911

[74] Rotinen M. ONECUT2 is a targetable master regulator of lethal prostate cancer that suppresses the androgen axis. Nat. Med. 24, 1887–1898 (2018). 30478421 10.1038/s41591-018-0241-1 PMC6614557

[75] Zhao Z.-H., Meng T.-G., Gao F., Schatten H. & Sun Q.-Y. Spatiotemporal and single-cell atlases to dissect cell lineage differentiation and regional specific cell types in mouse ovary morphogenesis. 2023.07.21.549985 Preprint at 10.1101/2023.07.21.549985 (2023). PMC12134226 40461746

[76] Ragvin A. Long-range gene regulation links genomic type 2 diabetes and obesity risk regions to HHEX, SOX4, and IRX3. Proc. Natl. Acad. Sci. U. S. A. 107, 775–780 (2010). 20080751 10.1073/pnas.0911591107 PMC2818943

[77] Fu A. IRX3 and IRX5 collaborate during ovary development and follicle formation to establish responsive granulosa cells in the adult mouse†. Biol. Reprod. 103, 620–629 (2020). 32507881 10.1093/biolre/ioaa100 PMC7822710

[78] Lamolet B. A pituitary cell-restricted T box factor, Tpit, activates POMC transcription in cooperation with Pitx homeoproteins. Cell 104, 849–859 (2001). 11290323 10.1016/s0092-8674(01)00282-3

[79] Corradin O. Combinatorial effects of multiple enhancer variants in linkage disequilibrium dictate levels of gene expression to confer susceptibility to common traits. Genome Res. 24, 1–13 (2014). 24196873 10.1101/gr.164079.113 PMC3875850

[80] Kulakovskiy I. V. HOCOMOCO: towards a complete collection of transcription factor binding models for human and mouse via large-scale ChIP-Seq analysis. Nucleic Acids Res. 46, D252–D259 (2018). 29140464 10.1093/nar/gkx1106 PMC5753240

[81] Rauluseviciute I. JASPAR 2024: 20th anniversary of the open-access database of transcription factor binding profiles. Nucleic Acids Res. gkad1059 (2023) doi:10.1093/nar/gkad1059. PMC10767809 37962376

[82] Bailey T. L., Johnson J., Grant C. E. & Noble W. S. The MEME Suite. Nucleic Acids Res. 43, W39–W49 (2015). 25953851 10.1093/nar/gkv416 PMC4489269

[83] Hudaiberdiev S. Modeling islet enhancers using deep learning identifies candidate causal variants at loci associated with T2D and glycemic traits. Proc. Natl. Acad. Sci. U. S. A. 120, e2206612120 (2023). 37603758 10.1073/pnas.2206612120 PMC10469333

[84] Luo Y. New developments on the Encyclopedia of DNA Elements (ENCODE) data portal. Nucleic Acids Res. 48, D882–D889 (2020). 31713622 10.1093/nar/gkz1062 PMC7061942

[85] Amemiya H. M., Kundaje A. & Boyle A. P. The ENCODE Blacklist: Identification of Problematic Regions of the Genome. Sci. Rep. 9, 9354 (2019). 31249361 10.1038/s41598-019-45839-z PMC6597582

[86] Grant C. E., Bailey T. L. & Noble W. S. FIMO: scanning for occurrences of a given motif. Bioinformatics 27, 1017–1018 (2011). 21330290 10.1093/bioinformatics/btr064 PMC3065696

[87] Rauluseviciute I. JASPAR 2024: 20th anniversary of the open-access database of transcription factor binding profiles. Nucleic Acids Res. 52, D174–D182 (2024). 37962376 10.1093/nar/gkad1059 PMC10767809

[88] Vorontsov I. E. HOCOMOCO in 2024: a rebuild of the curated collection of binding models for human and mouse transcription factors. Nucleic Acids Res. 52, D154–D163 (2024). 37971293 10.1093/nar/gkad1077 PMC10767914

[89] Hazell Pickering S., Abdelhalim M., Collas P. & Briand N. Alternative isoform expression of key thermogenic genes in human beige adipocytes. Front. Endocrinol. 15, 1395750 (2024). 10.3389/fendo.2024.1395750 PMC11163967 38859907

[90] Weis-Banke S. E. Mutant FOXL2C134W Hijacks SMAD4 and SMAD2/3 to Drive Adult Granulosa Cell Tumors. Cancer Res. 80, 3466–3479 (2020). 32641411 10.1158/0008-5472.CAN-20-0259 PMC8278322

[91] Kent W. J., Zweig A. S., Barber G., Hinrichs A. S. & Karolchik D. BigWig and BigBed: enabling browsing of large distributed datasets. Bioinformatics 26, 2204–2207 (2010). 20639541 10.1093/bioinformatics/btq351 PMC2922891

[92] Zhang Y. Model-based Analysis of ChIP-Seq (MACS). Genome Biol. 9, R137 (2008). 18798982 10.1186/gb-2008-9-9-r137 PMC2592715

[93] Liberzon A. Molecular signatures database (MSigDB) 3.0. Bioinformatics 27, 1739–1740 (2011) 21546393 10.1093/bioinformatics/btr260 PMC3106198

[94] Szklarczyk D. The STRING database in 2023: protein-protein association networks and functional enrichment analyses for any sequenced genome of interest. Nucleic Acids Res. 51, D638–D646 (2023). 36370105 10.1093/nar/gkac1000 PMC9825434

