# Supplementary material for: Regulatory risk loci link disrupted androgen response to pathophysiology of Polycystic Ovary Syndrome
Source: medRxiv. 2025 Mar 27:2025.03.26.25324630. Preprint. [Version 1] doi: 10.1101/2025.03.26.25324630 (PMC11974941; doi:10.1101/2025.03.26.25324630)
Supplement: Supplementary file 1 [file media-2.pdf]

Supplementary Information for

Regulatory risk loci link disrupted androgen response to pathophysiology of Polycystic Ovary Syndrome

Jaya Srivastava and Ivan Ovcharenko*

Division of Intramural Research, National Library of Medicine, National Institutes of Health, Bethesda, MD, 20892, USA.

* To whom correspondence should be addressed: ovcharen@nih.gov

This PDF file includes:

Supplementary figures S1 to S9

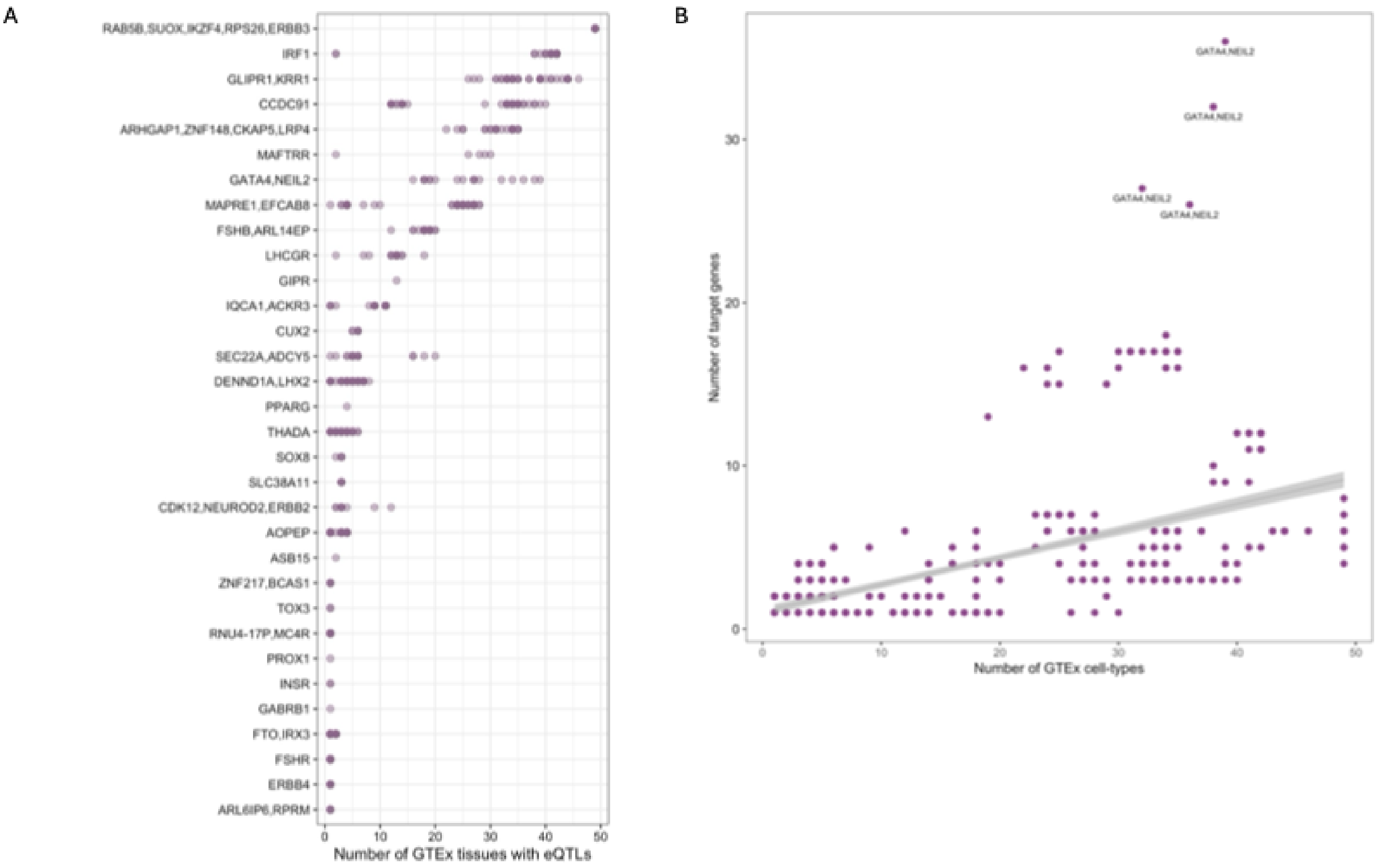

Figure S1: (A) Number of eQTLs overlapping with pcosSNVs in risk loci, (B) Correlation between the number of target genes regulated by eQTLs and the number of cell-types in which target gene(s) expression is affected.

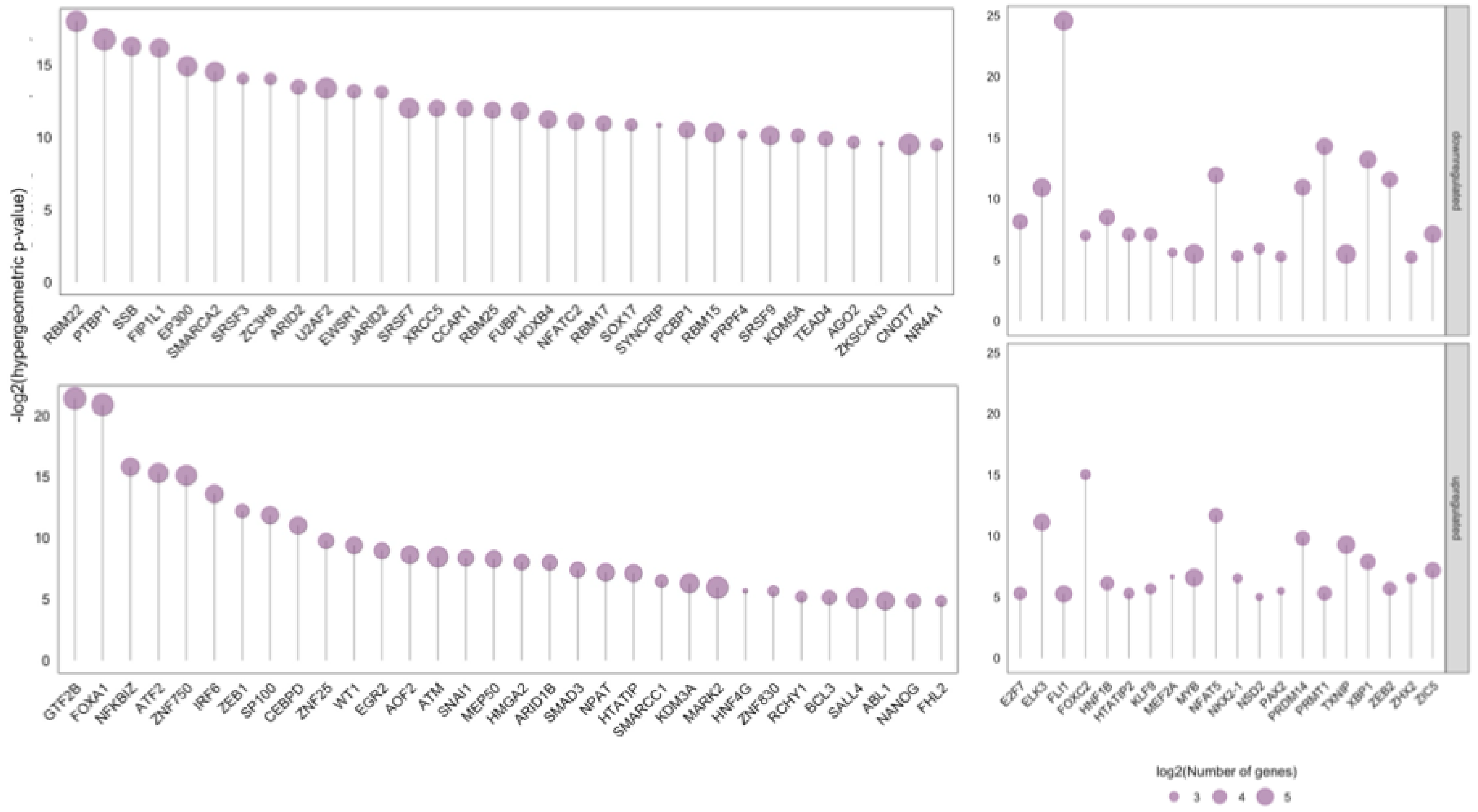

Figure S2: TFs leading to differential gene expression in PCOS patients in granulosa cells, identified using enrichment analysis pipeline of KnockTF2.0. Panels on the left indicate TFs significantly associated with downregulation (top) and upregulation (bottom) of gene expression. Panel on the right denote TFs associated with both up- and down-regulation.

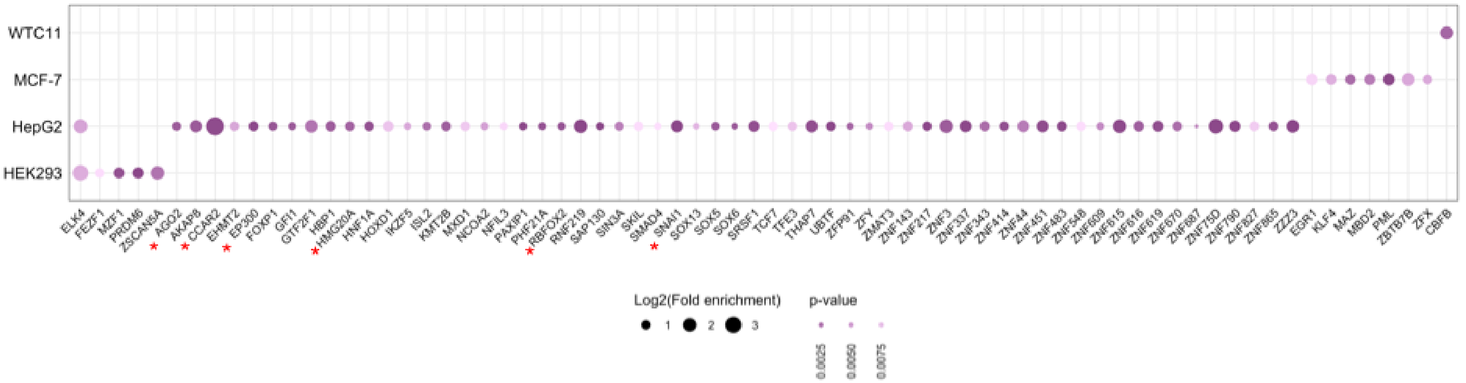

Figure S3: TFs whose ChIP-Seq peaks are enriched in pcosSNVs in the cell-types indicated on y-axis. Asterisks denote TFs identified in Figure S2.

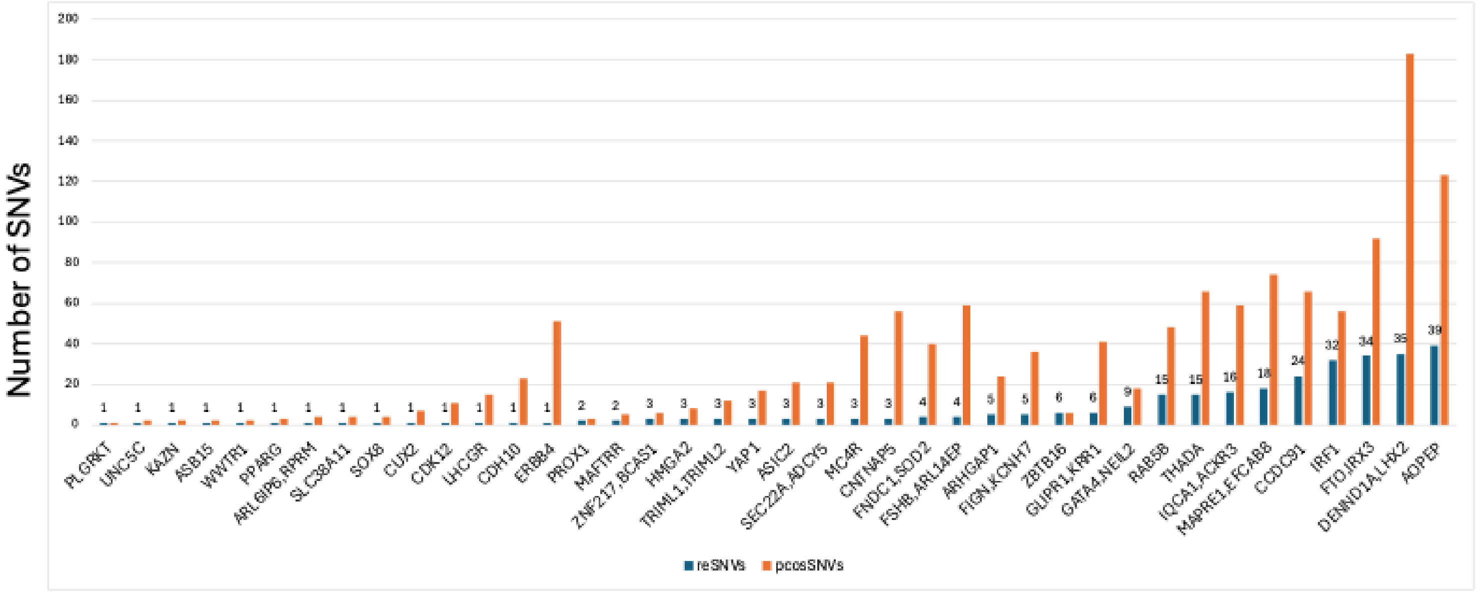

Figure S4: A comparison of number of pcosSNVs and reSNVs prioritized by TREDNet.

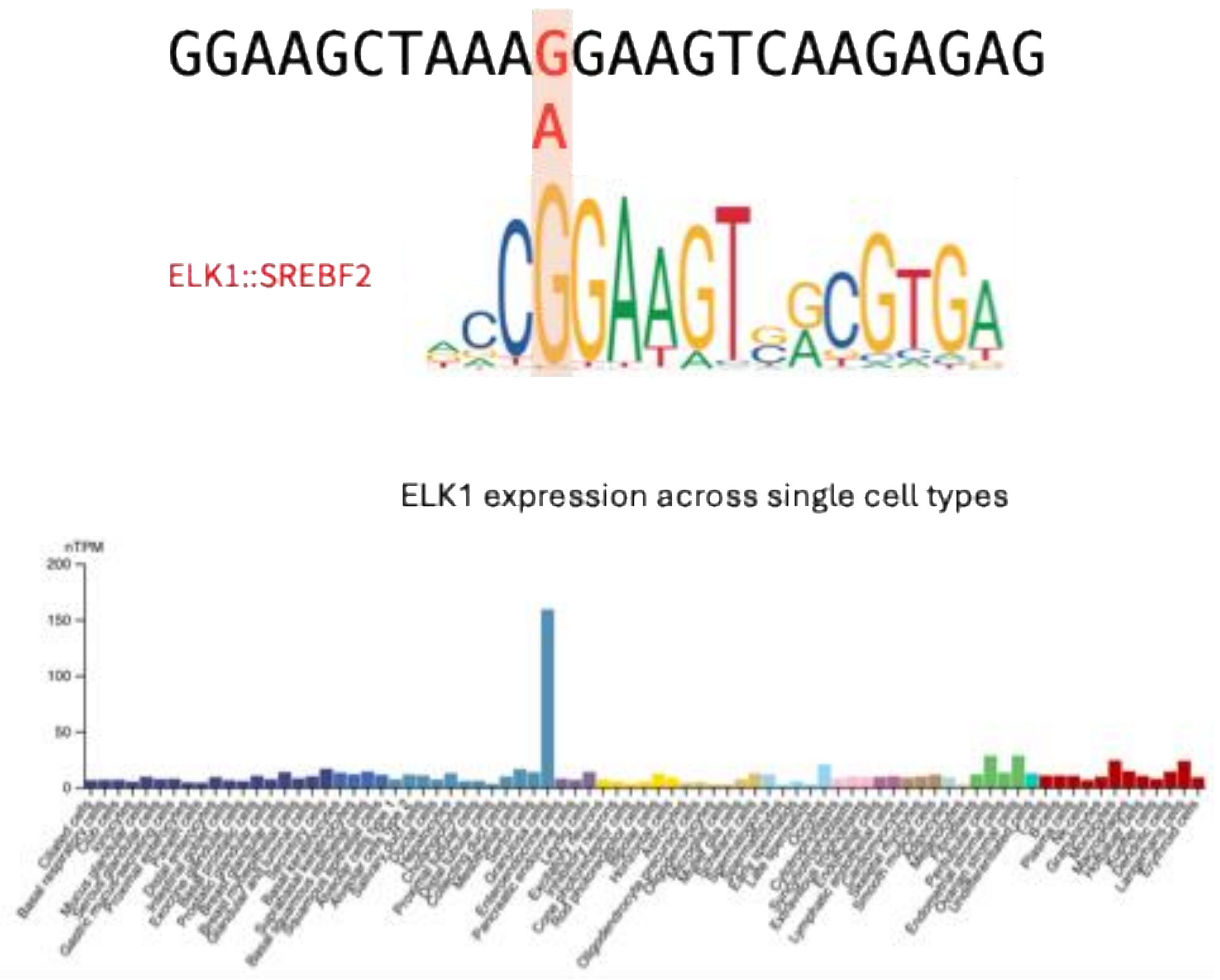

Figure S5: The G → A variant (rs113168128) in ERBB4 locus is predicted to disrupt the binding site of ELK1:SREBF2 motif (Top); Gene expression of ELK1 across single cells obtained from the Human Protein Atlas.

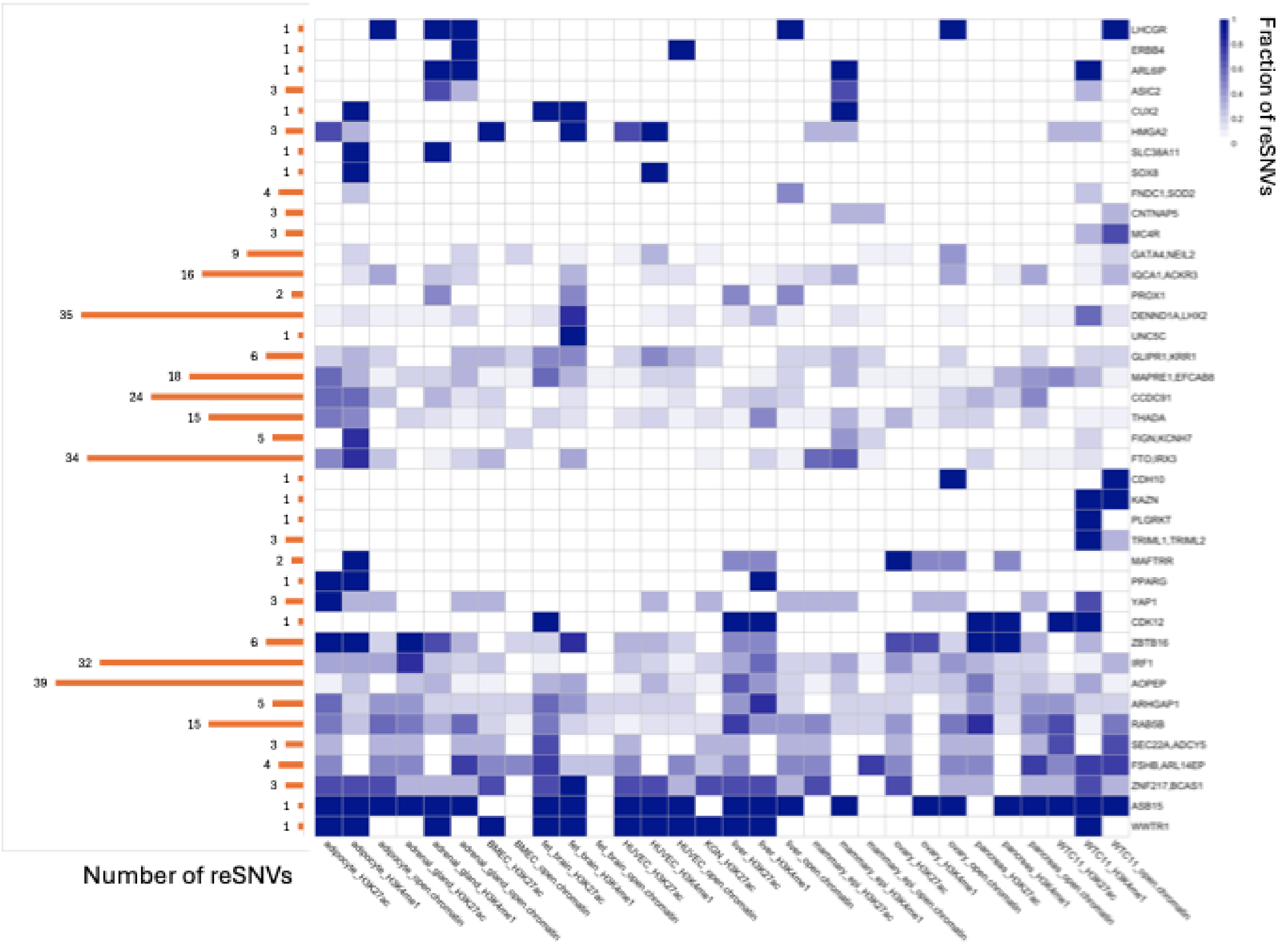

Figure S6: Heatmap depicting the overlap of reSNVs in the PCOS risk loci active regulatory regions of eleven cell-types. Total number of reSNVs predicted by TREDNet in each locus are indicated as bar plot on the left.

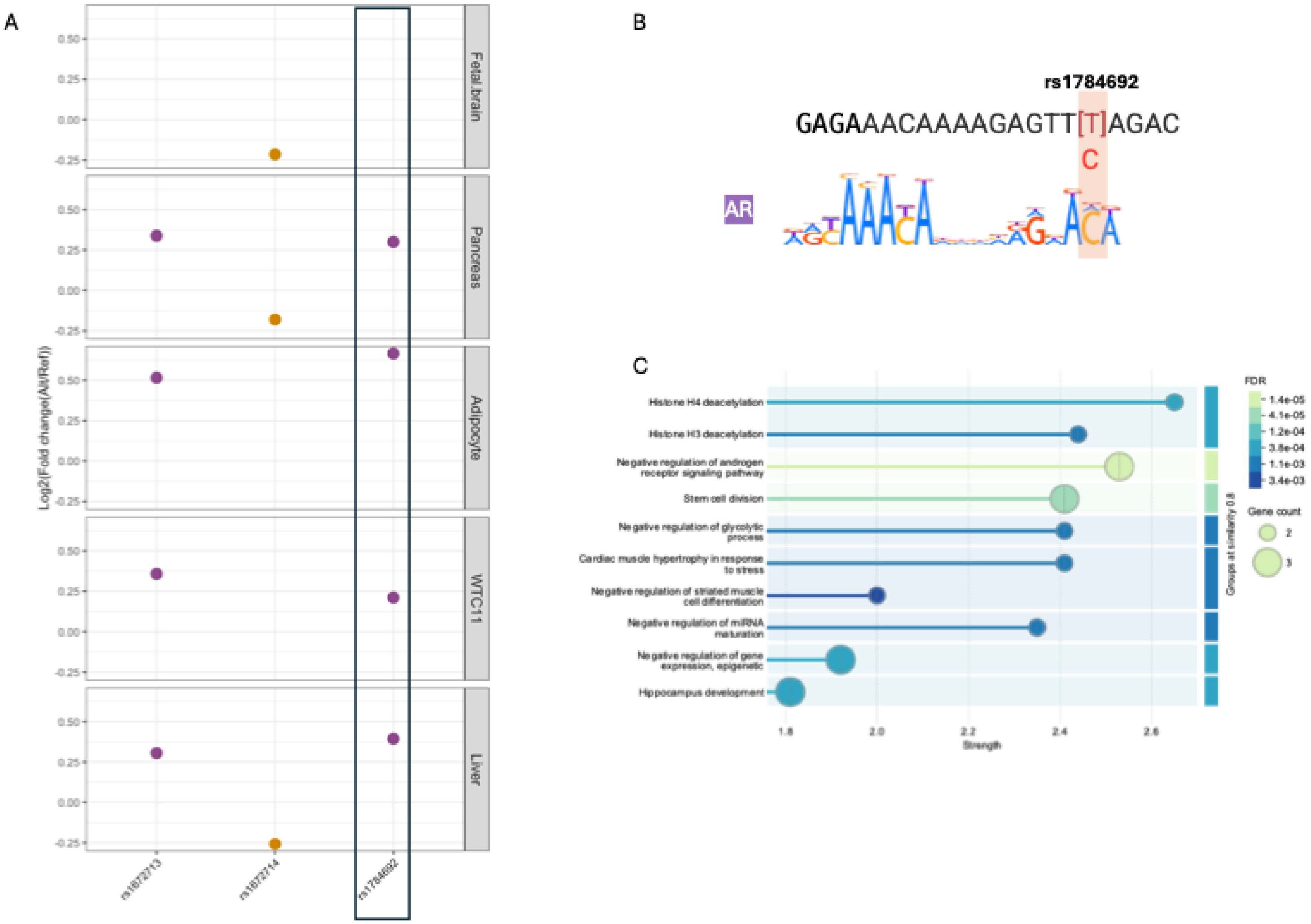

Figure S7: (A) Change in enhancer activity between reference and alternate alleles of reSNVs predicted by TREDNet in the *ZBTB16* locus. Purple and orange dots indicate enhancer strengthening and disrupting mutations, respectively, (B) rs1784692 is predicted to disrupt the binding site of AR, and (C) Biological processes associated with ZBTB16 based on its protein-protein interactions obtained from STRING database.

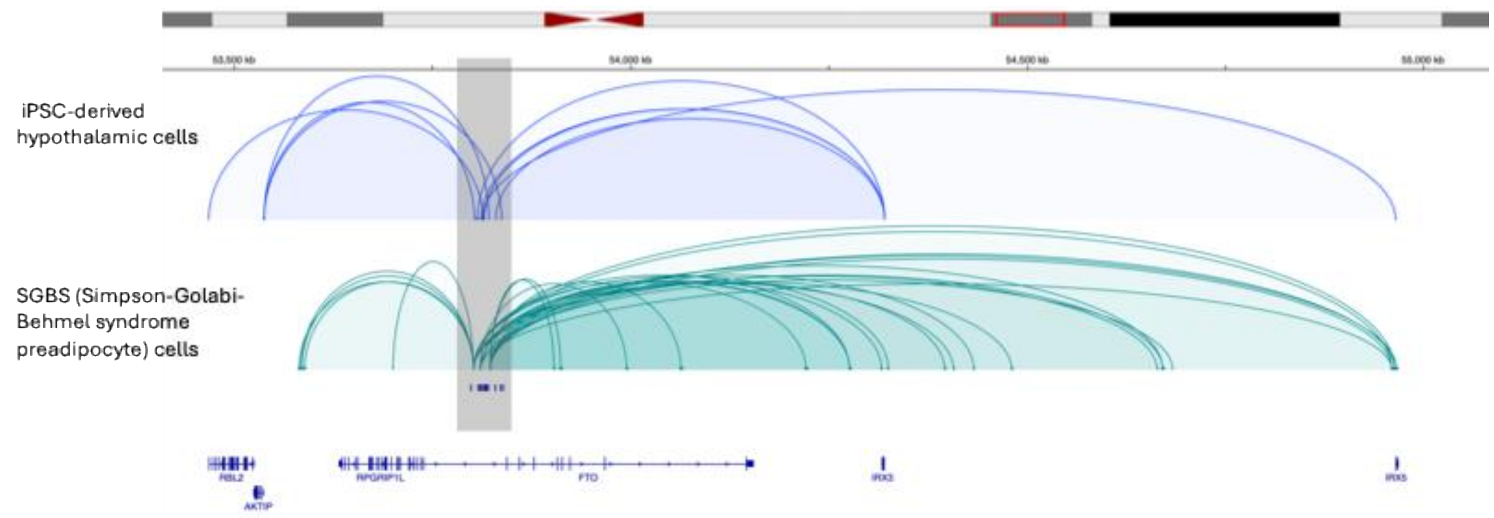

Figure S8: Hi-C based interactions between reSNVs of FTO locus (grey bar) with IRX3 and IRX5 in hypothalamic neurons and pre-adipocytes obtained from ref. 71.

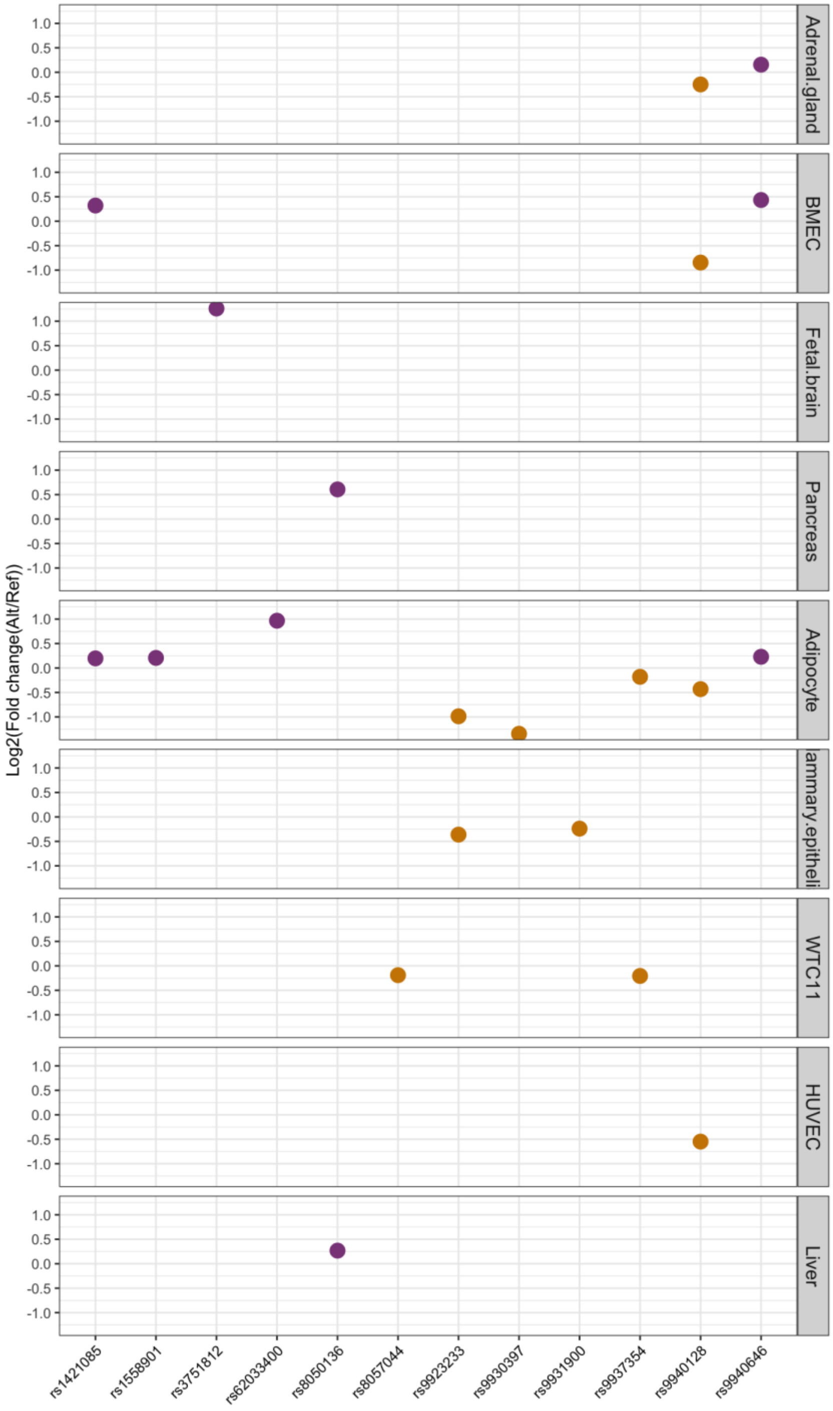

Figure S9: Change in enhancer activity between reference and alternate alleles of reSNVs predicted by TREDNet in the FTO locus. Purple and orange dots indicate enhancer strengthening and disrupting mutations, respectively.
